# Physicochemical and Sensory Characteristics of Spreadable Liver Pâtés with Annatto Extract (*Bixa orellana* L.) and Date Palm Co-Products (*Phoenix dactylifera* L.)

**DOI:** 10.3390/foods6110094

**Published:** 2017-10-29

**Authors:** Ana María Martín-Sánchez, Gelmy Ciro-Gómez, José Vilella-Esplá, José Ángel Pérez-Álvarez, Estrella Sayas-Barberá

**Affiliations:** 1 IPOA Research Group (UMH-1 and REVIV-Generalitat Valenciana), AgroFood Technology Department, Escuela Politécnica Superior de Orihuela, Universidad Miguel Hernández, Crta. Beniel km 3,2, E-03312 Orihuela, Alicante, Spain; ana.martin@umh.es (A.M.M.-S.); jvilella.dr@gmail.com (J.V.-E.); ja.perez@umh.es (J.Á.P.-Á.); 2 Faculty of Pharmaceutical and Food Science, University of Antioquia, AA 1226. Calle 67, No. 53-108, 050034 Medellín, Colombia; gelmyciro@gmail.com

**Keywords:** pâté, date co-products, annatto, physicochemical properties, emulsion stability, sensory properties

## Abstract

Two novel ingredients were incorporated into spreadable liver pâtés to study their effect on physicochemical and sensory characteristics and their possible use in the meat industry. Fresh date (*Phoenix dactylifera*, cv. Confitera) co-products, as a paste (0%, 2.5% and 7.5%), and annatto (*Bixa orellana*) extract (0 and 128 mg/kg), as a colourant, and their combinations were incorporated into liver pâtés to study their effect on the final quality. The six formulations were analysed for chemical composition, physicochemical characteristics (pH, a_w_, colour, emulsion stability, and texture), and sensory properties. Pâtés tolerated suitable incorporation of date paste, providing emulsifying activity and being able to counteract to some extent the emulsion destabilisation caused by the annatto. All formulations showed an acceptable sensory quality, particularly pâtés with annatto and 7.5% date paste, which was softer, juicier, and presented redness values similar to the control as well as better emulsion stability. The combined use of these novel ingredients could be used as natural ingredients.

## 1. Introduction

Fruits and vegetables are good sources of valuable compounds (fibre, phytochemicals, vitamins, etc.), which are also present in agri-food co-products and by-products [1]. Value could be added to these co-products by converting them into intermediate food ingredients. The most common fruit ingredients added to meat products are extracts and fibre concentrates [2], but the extraction of specific compounds needs to be considered in some cases due to the production of insufficient amounts and cost efficiency. So, a more challenging application would be the incorporation of whole fruit co-products for a better exploitation of the resources. Date palm (*Phoenix dactylifera* L.) is widely cultivated in some Mediterranean regions. In Europe, a major date palm grove is located in the South-East of Spain (Elche). The weather conditions affect date ripening, and bunches are collected having fruits in different ripening stages, including immature fruits. These “low-grade dates” are discarded, despite being rich in valuable compounds and bioactive substances [3]. 

The use of date co-products in meat products may lead to economic benefits to the date industry as well as the improvement of meat product properties. This is the case for pâté, known for its high fat and iron content and low level of antioxidants, and presents an adequate matrix to incorporate natural antioxidants [4]. In fact, trials in a campagne pork liver pâté indicated that dates could be properly incorporated, with small colour changes [5]. The use of annatto as a natural colourant may aid in achieving a more attractive colour. Annatto is a natural colourant derived from the seeds of a tropical bush (*Bixa orellana* L.), with a yellow-orange-red colour due to its high content of carotenoids, mainly bixin. It has antimicrobial and antioxidant activity, as it is a vital source of phenolic and flavonoid compounds [6]. Thus, this study aimed to evaluate the effect of adding date palm co-products and annatto extract, as a colourant, on the physicochemical and sensory characteristics of spreadable pork liver pâtés.

## 2. Materials and Methods 

### 2.1. Preparation of the Date Paste (DP)

Non-commercial fresh dates (*Phoenix dactylifera* L., cv. Confitera) from Elche (Spain) were collected at the “khalal stage” (not fully ripe, hard texture, yellow colour, astringent, and pH: 6.01). They were washed and frozen in vacuum pouches at −30 °C. The date paste (DP) was prepared following the procedure previously detailed by Martín-Sánchez et al. [5], which consisted of a blanching step followed by pitting, peeling, and blending in a cutter. This date variety and ripening stage was selected because it showed the highest antioxidant activity, good technological properties, and low sugar content [3]. 

### 2.2. Preparation of Annatto Extract (AE)

The annatto seeds, collected in San Luis (Antioquia, Colombia), were oven dried at 37 ± 0.2 °C for 48 h, and extracted with 95% ethanol (1:2; *w*/*v*) for 48 h at 4 °C. This extract was evaporated using a rotary evaporator R-205 (Büchi, Flawil, Switzerland) under reduced pressure at 40 °C. The obtained dry extract was the final annatto extract (AE), which was stored in amber bottles at 4 ± 0.2 °C. The properties of this AE were previously reported by Viuda-Martos et al. [6].

### 2.3. Manufacturing of Spreadable Type Pork Liver Pâtés

Pâtés were manufactured according to the traditional formula, with some modifications to adapt the product to the new ingredients: 65% pork jowl, 25% pork liver, and 10% backfat. The rest of ingredients (% related to meat ingredients) were: 8% whole egg, 2% potato starch (PROSUR, Murcia, Spain), 1.8% salt, 1.5% caseinate (PROSUR, Murcia, Spain), 0.2% polyphosphates blend (PROSUR, 0.2% white pepper, 0.05% sodium ascorbate (PROSUR), 0.03% thyme, 0.03% garlic powder, 0.03% nutmeg, and 125 mg/kg sodium nitrite (PROSUR). Pâtés were divided into six treatments, representing the concentrations of DP (0%, 2.5%, and 7.5%) and AE (0 and 128 mg/kg) chosen based on previous experiments. The chopped liver was soaked for 15 min in cold water, the jowl was scalded at 100 °C for 10 min, and the AE (water-insoluble) was mixed with the backfat. All the ingredients were comminuted in a cutter (Tecator 1094 Homogeneizer, Tekator, Höganäs, Sweden) until the formation of the emulsion, and the mixture was stuffed into artificial casings Fibran-Pack (Fibran, Girona, Spain) with a diameter of 5 cm and a length of 10–15 cm (400 g/piece), clipped at both ends and heated in hot water (95 °C) until the core reached 72 °C. After cooling, the samples were stored overnight (3 ± 1 °C and 85% RH (Relative Humidity)). The following types of pâtés were fabricated: control pâté; DP-2.5 (pâté with 2.5% date paste); DP-7.5 (pâté with 7.5% date paste); AE (pâté with 128 mg/kg annatto extract); DP-2.5+AE (pâté with 2.5% date paste and 128 mg/kg annatto extract); and DP-7.5+AE (pâté with 7.5% date paste and 128 mg/kg annatto extract). Samples from each treatment were taken for analyses on the day after the elaboration. These elaborations were independently conducted two times.

### 2.4. Pâté Chemical Composition

Moisture, ash, protein, and fat content were determined according to Official Methods [7]. Total dietary fibre (TDF) was identified in the defatted samples by the enzymatic-gravimetric AOAC method 991.43 [8]. The residual nitrite level (mg/kg) was determined by following the standard ISO 2918 [9]. Iron analyses were carried out through the ferrozine assay [10]. Three replicates were carried out for each chemical component (*n* = *3*).

### 2.5. pH and Water Activity (a_w_)

The pH was determined with a pH-meter (Model 507, Crison Instruments S.A., Barcelona, Spain) equipped with a combined electrode for solids (Cat. No. 52, Crison Instruments S.A., Barcelona, Spain), which was inserted into six different parts of the pâtés (*n* = *6)*. Water activity was measured at 25 °C in a Novasina TH-500 (Axair Ltd., Pfaeffikon, Switzerland) in duplicate (*n* = *2)*. 

### 2.6. Colour Determinations

The CIELAB colour space (L*: lightness; a*: redness; b*: yellowness), chroma (C*), and hue (*h*°) values were obtained using a spectrophotometer (Minolta CM-2600 (Minolta Camera Co. Osaka, Japan), illuminant D_65_, 10° observer, SCI mode, 11 mm aperture, and 8 mm for measurement) with a spectrally pure glass (CR-A51, Minolta Camera Co. Osaka, Japan) between the samples (*n* = 9) and the equipment. The total colour difference between the control and the other treatments was calculated by: ∆E* = ((∆L*)^2^ + (∆a*)^2^ + (∆b*)^2^)^1/2^. 

### 2.7. Emulsion Stability and Texture Analysis

The emulsion stability was measured following the procedure used by Martin et al. [11]. Samples were evaluated at 4 and 25 °C in duplicate (*n* = 2), and the percentage of total expressible fluid (%TEF) separated from the pâtés was determined as follows: %TEF = ((Weight of tube with sample—Weight of tube with pellet)/Sample Weight)×100.

Texture profile analysis (TPA) was performed with a Texture Analyser TA-XT2i (Stable Micro Systems, Surrey, England). Pâté samples (*n* = 6) from each treatment were cut into cubes (1 × 1 × 1 cm) when cold, and they were subjected to a two-cycle compression to 80% original height with a speed of 5 mm/s immediately after cutting and at 20–25 °C. Cohesiveness, hardness, springiness, gumminess, and resilience were estimated [12].

### 2.8. Sensory Evaluation of Pâtés

A 35-member panel from the Miguel Hernández University evaluated the pâtés. The analyses were performed according to the required specifications [13]. Pâtés were served at room temperature, and unsalted crackers and knives were provided to spread the samples. The assessors evaluated the intensity of different attributes on a scale from 0 to 10: colour (extremely light–extremely dark), aroma (imperceptible–extremely intense), hardness when spreading the sample (extremely soft–extremely tough), cohesiveness (extremely disintegrated–extremely cohesive), juiciness (extremely dry–extremely moist), particle detection (imperceptible–extremely perceptible), and sweet–salty taste (extremely sweet–extremely salty). At the end of the test, panellists were asked to give a score for overall acceptability from 0 to 10.

### 2.9. Statistical Analysis

ANOVA analysed the results using SPSS 23.0 (IBM, SPSS Statistical Software, Inc., Chicago, IL, USA). Tukey’s Multiple Range test was used for a comparison of means. The statistical significance was expressed at *p* < 0.05.

## 3. Results and Discussion

### 3.1. General Composition of Pâtés

The results in Table 1 show that the addition of DP increased the moisture and fibre content, and reduced the protein, fat, and nitrite content. These variations were expected due to the dilution effect of the DP. Moisture values, around 40%, were lower than usually reported in this type of product (>45%) [14]; this could be due to differences between the ingredients used in the elaboration of the pâtés. The nitrite and iron content were also slightly reduced by the dilution effect of the DP. The presence of AE did not affect the proximate composition of pâtés (*p* > 0.05) significantly. The iron content increase of up to 4 µg/g could be partially justified by the iron content in the *Bixa orellana* seeds [15]. The combined use of DP-7.5 and AE improved iron content and fibre, and fat content was reduced; this represents a positive effect on nutritional properties, intending to enhance the image of meat products.

### 3.2. pH and a_w_ of Pâtés

The results in Table 2 show that the incorporation of DP tended to slightly decrease the pH, while the addition of AE did the opposite; however, all these variations are of little relevance, with no technical implications. Moreover, the values obtained (6.39–6.51) are in a normal range for this kind of product [14]. The addition of DP and/or AE increased the a_w_, especially when both DP and AE were combined. This may be related to the water incorporated by the DP, as well as some modifications that the AE could be exerting in the pâté structure.

### 3.3. Colour Characteristics of Pâtés

The colour parameters were affected by the addition of the new ingredients (Table 2). For example, 2.5% DP and the presence of AE with or without DP reduced L* (*p* < 0.05). When AE was added, the changes in L* were due to the colourant effect, hiding the effect of the DP. Redness values decreased proportionally to the DP content (*p* < 0.05), while the pâtés with AE, being very rich in carotenoids [6], led to a redder product. Nevertheless, this effect was only observed in AE and DP-2.5+AE pâtés, because when AE was combined with 7.5% DP a similar redness to the control pate was obtained. The DP and/or AE increased b*. This increase may be due to the yellow carotenoid lutein present in the DP at this stage of ripening [3]. Likewise, annatto is known to give a yellowish colour. Chroma showed that the addition of DP gave a colour intensity similar to that of the control, whereas all treatments with AE had higher C* values than the control. Hue indicates the degree of change of the colour from red (low *h*° values) to yellow (high *h*° values), revealing the results that the addition of DP and AE changed the colour of pâtés from red to a more yellowish colour, but tasters positively evaluated this effect as a less dark colour (Table 4, see Sensory properties). The ∆E* results pointed to evident visual differences between the control and the pâtés containing DP; while the addition of AE further increased ∆E*, these differences were not perceived the same way by the panel, who assessed the pâtés with DP alone with lower scores (Table 4). 

### 3.4. Emulsion Stability

In pâtés at 4 °C, the exudates, as expected, were lower than in those analysed at 25 °C (Table 3). At storage conditions, %TEF did not differ (*p* > 0.05) between the control and DP-7.5, while using 2.5% DP increased the stability (*p* < 0.05). However, the incorporation of AE significantly increased the separation of fluid, giving a more unstable emulsion regardless of the DP content. At 25 °C, the emulsion stability was improved when DP was added, but in combination with AE this enhancing effect was not observed except at a concentration of 7.5% DP. Therefore, the addition of DP greatly improved the emulsion stability at 25 °C. The unique drawback of adding AE would be the destabilisation of the emulsion to some extent, probably due to water and fat separation from the protein matrix. In fact, pâtés with AE only, at higher concentrations, presented a higher visual level of structural disintegration, as a granulated product (visible fat-like components). However, the addition of DP gave a more homogenous meat matrix, and all the ingredients were visually integrated. Other studies on the effect of various fibre-rich ingredients on emulsion meat stability concluded that these ingredients improve hydration properties and fat-holding capacity, reducing fat and water losses while increasing stability [16]. Along with this, the interactions between proteins and carbohydrates can help stabilise the emulsions [17]; likewise, some phenolic compounds may be able to interact with proteins and stabilise the emulsion structure [18]. However, AE interacted in some way with the pâté ingredients leading to the destabilisation of the emulsion. A possible explanation could be related to some of the phytochemicals present in AE, such as certain tannins [19], that are able to interact with proteins from the meat. Given that the pâtés were spreadable, the formula DP-7.5+AE could be highly acceptable since a good stability, better than the control at serving temperature, was obtained. 

### 3.5. Texture Profile Analysis

All textural parameters (Table 3) were affected by the addition of DP or/and AE. DP, alone or combined with AE, reduced hardness and gumminess (*p* < 0.05), yielding softer pâtés. The reduction in hardness was probably due to the incorporated water and fibre. In fact, differences in composition affect the protein:fat:water ratio, which is a significant factor for the resulting gel consistency. Furthermore, the fibre may disrupt the protein-water or protein-protein gel network, decreasing the gel strength of the pâté [20]. According to Estévez et al. [21], reduced hardness may also be related to better emulsion stability, which agrees with the lower %TEF found in pâtés with DP. The addition of DP (2.5% and 7.5%) increased springiness and cohesiveness (7.5%). Resilience slightly increased in DP-2.5 and DP-7.5 pâtés, but the treatment with AE did not show differences regarding the control. Therefore, the addition of DP resulted in a better emulsion and textural properties of this spreadable pâté.

### 3.6. Sensory Properties

Panellists detected significant differences (*p* < 0.05) for colour, taste, hardness, and juiciness (Table 4). The addition of DP led to lighter, sweeter, softer, and juicier pâtés than the control, particularly at 7.5% DP. When DP was combined with AE, the colour differences were lower compared with the control. Sweetness increased parallel to the DP content, while AE did not influence this taste perception. Panellists were able to distinguish the DP content according to the colour intensity, and increasing DP concentrations led to lower scores. Although DP lightened the colour, in combination with AE, the DP-added pâtés obtained similar values to the control so that no colour differences could be detected visually among them. Hardness values agreed with the instrumental test, being softer for those with DP. The control was perceived as the driest one, so that juiciness increased with the addition of DP and/or AE. The particle detection increased due to the AE, but in combination with DP, the score was the same as that for the control. This confirms the effect of AE on the matrix form of the product, giving a more granulated structure. Regarding the overall preference, no significant differences were found among the six pâtés, although the highest average score was attained by the pâté containing AE and 7.5% DP. This result is of great interest since it indicates that a high content of DP can be incorporated together with AE with no detrimental effects on the sensory aspects.

## 4. Conclusions

This study shows the possibility of incorporating these two new ingredients in pâté. The decrease in a* caused by the date paste is offset by the combination of annatto extract, as a colourant, improving redness. Date paste is greatly useful in improving emulsion stability and texture. Therefore, in the pâtés with 7.5% date paste and annatto extract, the date paste would act as a natural emulsifier and annatto extract as a natural colourant, obtaining a highly accepted product by panellists: softer and juicier, with a similar colour to the control and the best emulsion stability (25 °C). The combined use of both ingredients improves product quality, which allows adding value to fresh date co-products that otherwise would be wasted and would increase environmental contamination. 

## Figures and Tables

**Table 1:** Chemical composition of the different pork liver pâtés with date paste (DP) (0%, 2.5%, and 7%) and annatto extract (AE) (0 and 128 mg/kg) (Mean ± Standard Error (SE)).

Parameters	Control	DP-2.5	DP-7.5	AE	DP-2.5+AE	DP-7.5+AE
Moisture (%)	40.11 ± 0.89 ^a^	42.37 ± 0.66 ^ab^	43.79 ± 0.38 ^b^	39.80 ± 0.79 ^a^	42.01 ± 0.99 ^ab^	42.69 ± 0.36 ^ab^
Fat (%)	38.05 ± 0.70 ^b^	36.81 ± 0.27 ^ab^	34.27 ± 0.64 ^a^	38.40 ± 0.61 ^b^	35.81 ± 0.70 ^ab^	34.83 ± 0.14 ^a^
Protein (%)	14.54 ± 0.07 ^bc^	13.47 ± 0.04 ^ab^	12.87 ± 0.04 ^a^	15.07 ± 0.30 ^c^	14.12 ± 0.29 ^bc^	13.84 ± 0.41 ^ab^
Ash (%)	2.06 ± 0.04 ^a^	2.07 ± 0.03 ^a^	2.13 ± 0.06 ^a^	2.14 ± 0.06 ^a^	2.15 ± 0.08 ^a^	2.17 ± 0.04 ^a^
Fibre (%)	0.67 ± 0.03 ^a^	0.88 ± 0.04 ^b^	1.38 ± 0.03 ^c^	0.67 ± 0.04 ^a^	0.87 ± 0.04 ^b^	1.39 ± 0.04 ^c^
Nitrite (mg/kg)	26.12 ± 0.54 ^bc^	25.21 ± 0.31 ^b^	23.54 ± 0.10 ^a^	27.39 ± 0.16 ^c^	27.14 ± 0.05 ^c^	25.18 ± 0.09 ^b^
Iron (µg/g)	40.05 ± 1.59 ^b^	36.54 ± 0.03 ^a^	34.86 ± 0.45 ^a^	44.60 ± 0.54 ^d^	43.71 ± 0.36 ^dc^	42.22 ± 1.61 ^c^

^a–d^ Different superscripts within the same row indicate differences between treatment groups (*p* < 0.05).

**Table 2:** Physicochemical parameters (pH, water activity, and colour) of the different pâtés with date paste (DP) (0%, 2.5% and 7%) and annatto extract (AE) (0 and 128 mg/kg) (Mean ± SE).

Parameters	Control	DP-2.5	DP-7.5	AE	DP-2.5+AE	DP-7.5+AE
pH	6.47 ± 0.01 ^b^	6.43 ± 0.01 ^b^	6.39 ± 0.01 ^a^	6.51 ± 0.01 ^bc^	6.50 ± 0.01 ^bc^	6.50 ± 0.01 ^bc^
a_w_	0.927 ± 0.001 ^a^	0.931 ± 0.001 ^b^	0.931 ± 0.001 ^b^	0.931 ± 0.001 ^b^	0.938 ± 0.001 ^c^	0.938 ± 0.001 ^c^
L*	57.3 ± 0.2 ^b^	55.3 ± 0.2 ^a^	57.2 ± 0.4 ^b^	55.0 ± 0.4 ^a^	54.4 ± 0.2 ^a^	54.5 ± 0.1 ^a^
a*	8.6 ± 0.1 ^c^	7.6 ± 0.1 ^b^	6.8 ± 0.2 ^a^	10.2 ± 0.2 ^d^	9.8 ± 0.1 ^d^	8.4 ± 0.1 ^c^
b*	11.2 ± 0.2 ^a^	12.5 ± 0.1 ^b^	11.9 ± 0.1 ^b^	13.5 ± 0.2 ^c^	13.9 ± 0.2 ^c^	13.9 ± 0.2 ^c^
C*	14.1 ± 0.2 ^ab^	14.6 ± 0.1 ^b^	13.7 ± 0.2 ^a^	16.9 ± 0.1 ^d^	17.0 ± 0.2 ^d^	16.3 ± 0.2 ^c^
*h*°	52.6 ± 0.4 ^a^	58.7 ± 0.4 ^c^	60.4 ± 0.7 ^c^	52.9 ± 0.7 ^ab^	54.9 ± 0.3 ^b^	59.1 ± 0.2 ^c^
*∆E*	0 ^a^	2.6 ± 0.2 ^b^	2.3 ± 0.2 ^b^	3.7 ± 0.3 ^c^	4.1 ± 0.2 ^c^	3.9 ± 0.2 ^c^

^a–d^ Different superscripts within the same row indicate differences between treatment groups (*p* < 0.05). L*: lightness; a*: redness; b*: yellowness; C*: chroma, *h*°: hue, *∆E:* total colour difference.

**Table 3:** Emulsion stability measured as total expressible fluid (% TEF) at 4 and 25 °C, and textural properties of the different pâtés with date paste (DP) (0%, 2.5%, and 7%) and annatto extract (AE) (0 and 128 mg/kg) (Mean ± SE).

Parameters	Control	DP-2.5	DP-7.5	AE	DP-2.5+AE	DP-7.5+AE
% TEF (4 °C)	1.8 ± 0.1 ^b^	0.6 ± 0.1 ^a^	1.3 ± 0.1 ^b^	3.7 ± 0.2 ^c^	3.5 ± 0.3 ^c^	3.4 ± 0.7 ^c^
% TEF (25 °C)	7.4 ± 0.3 ^c^	1.6 ± 0.1 ^a^	1.9 ± 0.1 ^a^	6.2 ± 0.6 ^c^	5.9 ± 0.3 ^bc^	4.3 ± 0.1 ^b^
Hardness (g)	3297 ± 114 ^b^	2404 ± 149 ^a^	2209 ± 150 ^a^	3609 ± 155 ^b^	2654 ± 128 ^a^	2530 ± 131 ^a^
Springiness (mm)	5.77 ± 0.07 ^a^	6.09 ± 0.06 ^bc^	6.09 ± 0.08 ^bc^	5.82 ± 0.05 ^ab^	5.74 ± 0.09 ^a^	6.12 ± 0.03 ^c^
Cohesiveness	0.23 ± 0.01 ^a^	0.23 ± 0.01 ^a^	0.31 ± 0.02 ^b^	0.22 ± 0.00 ^a^	0.22 ± 0.00 ^a^	0.24 ± 0.01 ^a^
Gumminess (g)	750.1 ± 19.5 ^bc^	555.0 ± 30.2 ^a^	690.7 ± 70.0 ^abc^	798.1 ± 26.9 ^c^	575.7 ± 33.1 ^ab^	605.9 ± 33.0 ^ab^
Resilience	0.15 ± 0.01 ^a^	0.18 ± 0.01 ^bc^	0.21 ± 0.01 ^c^	0.16 ± 0.00 ^ab^	0.15 ± 0.00 ^a^	0.17 ± 0.00 ^ab^

^a–c^ Different superscripts within the same row indicate differences between treatments (*p* < 0.05).

**Table 4:** Sensory evaluation of the different pâtés with date paste (DP) (0%, 2.5% and 7%) and annatto extract (AE) (0 and 128 mg/kg) (Mean ± SE).

Parameter	Control	DP-2.5	DP-7.5	AE	DP-2.5+AE	DP-7.5+AE
Colour (Light-Dark)	5.9 ± 0.3 ^bc^	4.8 ± 0.3 ^ab^	4.2 ± 0.2 ^a^	6.1 ± 0.4 ^c^	5.7 ± 0.3 ^bc^	5.4 ± 0.3 ^abc^
Aroma (Low-High)	5.4 ± 0.4	6.3 ± 0.4	5.8 ± 0.3	5.6 ± 0.4	6.2 ± 0.3	6.3 ± 0.2
Taste (Sweet-Salty)	6.4 ± 0.3 ^bc^	5.9 ± 0.3 ^bc^	4.5 ± 0.3 ^a^	6.8 ± 0.3 ^c^	5.3 ± 0.3 ^ab^	4.7 ± 0.2 ^a^
Cohesiveness (Low-High)	5.3 ± 0.3	4.8 ± 0.3	4.6 ± 0.2	5.4 ± 0.4	5.2 ± 0.3	5.4 ± 0.3
Hardness (Soft-Hard)	5.3 ± 0.2 ^ab^	4.5 ± 0.3 ^ab^	4.2 ± 0.3 ^a^	5.9 ± 0.3 ^b^	5.1 ± 0.4 ^ab^	4.5 ± 0.3 ^a^
Juiciness (Dry-Juicy)	4.9 ± 0.3 ^a^	6.1 ± 0.3 ^ab^	5.9 ± 0.4 ^ab^	6.1 ± 0.3 ^ab^	6.2 ± 0.3 ^b^	6.5 ± 0.3 ^b^
Particle detection (Low-High)	4.8 ± 0.3 ^a^	4.7 ± 0.4 ^a^	4.9 ± 0.4 ^a^	6.8 ± 0.3 ^b^	6.1 ± 0.5 ^ab^	5.3 ± 0.3 ^ab^
Overall preference	6.1 ± 0.3	5.8 ± 0.4	5.5 ± 0.4	6.6 ± 0.4	6.0 ± 0.5	7.0 ± 0.3

^a–c^ Different superscripts within the same row indicate differences between treatment groups (*p* < 0.05).
